# Being active when living within a large body: experiences during lifestyle intervention

**DOI:** 10.1080/17482631.2020.1736769

**Published:** 2020-03-10

**Authors:** Bente Skovsby Toft, Kathleen Galvin, Claus Vinther Nielsen, Lisbeth Uhrenfeldt

**Affiliations:** a Department of Lifestyle Rehabilitation, Horsens Regional Hospital, Brædstrup, Denmark; b School of Health Sciences, University of Brighton, Brighton, UK; c Department of Public Health, Aarhus University, Aarhus, Denmark; d Faculty of Nursing and Health Science, Nord University, Bodø, Norway

**Keywords:** Adults, experience, hermeneutics, life style, lifeworld, obesity, physical activity, qualitative study, well-being

## Abstract

**Background**: In-depth understanding of the experiences of both well-being and suffering in relation to being severely obese and becoming active through lifestyle intervention is lacking.

**Aim**: to explore and describe adults’ existential experiences of being active, when living within a large body—before and during a lifestyle intervention.

**Methods: **A longitudinal design of repeated indivi****dual interviews with 16 adults with BMI ≥40, based on hermeneutic phenomenology, existential philosophy and a theory of well-being was performed. The study was approved by the Danish health authorities.

**Results**: Two dimensions of experiences were found; “Living within a downward spiral” and “Striving for enjoyment and settlement”. The themes describing suffering were: ‘Sense of being thwarted and defeated ‘ and “Tackling energy depletion and impact of sense of self”. The themes describing well-being were: “Hoping for renewal and energised resoluteness” and “Enduring discomfort and feeling safe”.

**Conclusions**: Interacting existential experiences can be facilitators or barriers for physical activity. It seems relevant for health care providers to address the individual’s lifeworld experiences of well-being, lack of well-being and suffering. Well-being as a sense of feeling “at home” when physically active may break down an inactivity spiral. Promoting well-being is a legitimate aim of lifestyle intervention.

## Introduction

There is a general lack of understanding regarding the perspectives of severely obese adults in obesity-related research, particularly when designing health interventions. Further, being severely obese and becoming active through lifestyle intervention may be associated with experiences of both well-being and suffering.

This study is concerned with linking severe obesity and health to the existential experiences of being active during lifestyle intervention. In activity, severely obese individuals may describe different existential experiences, which may provide insights regarding physical activity (PA) interventions.

This study is based on data generated at a national centre for lifestyle intervention, at a public Danish hospital. In an existential understanding, lifestyle change combines the striving for “becoming”, i.e., being able to act and do meaningful things and to live “authentically” with being content, i.e., finding settlement with one’s current situation (Heidegger, 1962/2002). Well-being, in a philosophical understanding, is a value-laden and holistic way of being well as everyday life unfolds (Sarvimäki, 2006). Well-being is often a hidden and unreflected experience (Galvin & Todres, 2011); however, obesity may disturb well-being by adding discomfort and physical limitations (Dahlberg & Segesten, 2010). This may bring a feeling of not being “at home” in one’s own body and/or in relation to PA (Heidegger, 1962/2002).

## Background

Obesity is commonly classified by Body Mass Index (BMI). Obesity grade III, i.e., ≥40 kg/m^2^ is referred to as severe obesity in this study. This is chosen to use an ontological-based language rather than the bio-medical term “morbid obesity” (Nutter et al., 2016).

Severely obese individuals report lower self-rated health and psychological well-being than the rest of the population (Capodaglio et al., 2013) and they have a very low prevalence of exercise (Stubbs & Lavin, 2013). Being severely obese you may belong to a disadvantaged and marginalized group of people with different vulnerabilities, i.e., low self-esteem, low socio-economic status, and short education (WHO, 2014) as well as a more frequent prevalence of depression (Somerville et al., 2015) and impairments in psychosocial functioning (Capodaglio et al., 2013).

PA can be an important part of improving health for adults with severe obesity (Aadland & Robertson, 2012) and it is an integral part of any treatment plan due to the numerous health benefits that occur from PA even in the absence of weight loss (Swift et al., 2014). PA includes lifestyle activities, i.e., everyday tasks as well as structured exercise and is together with diet and behaviour therapy a part of multi-component lifestyle intervention programmes for obesity (Wadden et al., 2004).

PA is individually perceived as an embodied unreflected knowledge of what is felt as pleasant or unpleasant, possible or impossible (Ekkekakis & Lind, 2006; Wingo, 2010). Body size influences the experiences of being active (Groven & Engelsrud, 2010) and may also present barriers causing ambivalence and avoidance of being active (Bombak, 2015) due to feeling alienated and objectified (Lewis et al., 2011; Vartanian & Novak, 2011). In contrast, improvement in well-being and vitality are factors facilitating PA participation among obese individuals (Zabatiero et al., 2016) and well-being has been suggested as a longterm outcome when treating people with obesity (Peacock et al., 2014). This is due to the fact that a massive focus on weight loss may eventually lead to weight gain and inactivity (Owen-Smith et al., 2014; Vartanian & Novak, 2011). Emphasizing health through PA may be done by tailoring exercise prescriptions and instructions with sensitivity towards patients’ experiences (Bombak, 2014; Kahn et al., 2002), i.e., increase the possibility of enjoyment in exercises (Ekkekakis & Lind, 2006).

Different qualitative studies have explored severely obese individuals' experiences in relation to weight loss (Christiansen et al., 2007; Dahl et al., 2014; Thomas et al., 2008) or PA in relation to bariatric surgery (Dikareva et al., 2016; Peacock et al., 2014; Wiklund et al., 2011; Zabatiero et al., 2016). Recent studies have explored how living with obesity is experienced from an existential perspective (Haga et al., 2019a, 2019b; Ueland et al., 2019). However, less emphasis has been put on how PA is experienced by patients living with severe obesity and a systematic review found that very little is known about how facilitators and barriers to PA are lived through a process of lifestyle changed and described in relation to existential experiences of well-being (Toft & Uhrenfeldt, 2015). There is a need for a new understanding to inform health care providers (HCP) how it feels to be active when living within a large body.

## Aim

The aim of this study is to explore and describe adults´ existential experiences of being active, when living within a large body—before and during a lifestyle intervention.

The research question was: How do severely obese individuals experience physical activity before and during lifestyle intervention?

## Design, methodology and methods

The longitudinal design of this study (Balmer & Richards, 2017) is influenced by qualitative inquiry and based on hermeneutic phenomenology with the philosophy of the lifeworld, i.e., the ontological ideas of the human being by Martin Heidegger (Heidegger, 1962/2002). In addition, the methodology is supported by the hermeneutic thoughts of understanding by Hans Georg Gadamer (Gadamer, 2013).

In this study, the concept of lifeworld holds the meaningful nature of well-being in each individual’s everyday life. This “Being-in-the-world” is the lived and felt experiences between self and the world (Heidegger, 1962/2002). The lifeworld domains are spatiality, temporality, intersubjectivity, mood, identity and embodiment (Ashworth, 2003). They can occur during dwelling as a “letting-be-ness” with what has been given (Heidegger, 2001; Mugerauer, 2008), as well as during mobility characterized by possibilities in striving for `becoming` by means of who you are and what you are doing in life. Another person’s lifeworld can by captured by others through dialogue by getting concrete descriptions of specific lived happenings, implicit meanings and essences of their lifeworld experiences (Dahlberg et al., 2009).

Moreover, the study is founded upon Gadamer’s hermeneutic concepts of understanding and the fusion of horizons. This means that it is a prerequisite for the researcher to know something beforehand and to share an interest in a topic with the participants to achieve mutual understanding. Developing a mutual understanding takes place through language as a medium that bears its own truth within itself and allows something to “emerge” which henceforth existed (Gadamer, 2013, p. 402). Through interviews, the researcher puts the pre-understandings of the topic in a continuous circular back-and-forth process between parts and the wholes in order to expand previous understandings and to be open to possible alternative meanings. This hermeneutic process was applied to both conducting the interviews and analysing the data.

The lifeworld domains have served the researcher’s pre-understanding and have guided the data collection by structuring the interview guides and topics for investigation (Brinkmann & Kvale, 2014). A conceptual framework with different kinds of well-being (Galvin & Todres, 2011) and suffering has served as a lens to guide the existential dimensions of the lifeworld into the project in a sensitizing manner (Galvin & Todres, 1953–2013). The pre-understanding informed by phenomenological perspectives on well-being was used in order to understand the wholeness of human beings. The complex condition of being severely obese strongly pointed to an existential approach of exploring what it is like to be active (Lindberg et al., 2016).

### Setting, participants and sampling

The setting was the Department of Lifestyle Rehabilitation, an in-hospital group-based intervention led by a team of interdisciplinary HCPs, e.g., registered nurses, dieticians, registered physiotherapists, occupational therapists and cognitive psychologists. Each patient is hospitalized three times in health-promoting modules of 4 days during a 6-month period with telephone counselling in-between modules. Approximately 600 patients living with obesity (BMI>30), diabetes, cardiovascular disease or lung disease undergo this intervention each year. They are referred by a general practitioner.

Participants were purposefully recruited (Coyne, 1997) from a list of referred patients awaiting admission to the treatment programme. Twenty-three patients were contacted and invited to participate by a gatekeeper (Holloway & Galvin, 2016), i.e., the secretary of the department. Seven persons withdrew for different reasons: changed her mind after having read the information material, due to illness, anxiety and being unreachable at the time for appointment. Eight females and eight males were included according to the following criteria; age ≥18 years and BMI ≥40 kg/m^2^ and referred to lifestyle intervention. The females median BMI was 41,25 kg/m^2^ and their average age was 38 years. Males median BMI was 44,25 kg/m^2^ and the average age was 47 years (See Table I).

**Table I.** Details of age and BMI for participants (n = 16) entering the study

	Age		BMI	
Gender	Range	Median	Range	Median
Females	27-59	38*	40-48	41,25*
Males	25-68	47*	41-55	44,25*
Both	25-68	43,5**	40-55	43**

*median: mean values of observation 5 and 6 ** median: mean values of observation 8 and 9

The 15 of 16 participants had low- or middle-level education, 10 of the total participants were unemployed and 10 were living alone, mainly in smaller urban situated housings. Five participants had resident children (See Table II).

**Table II.** Characteristics of participants at the time of inclusion (n = 16)

#	Gender Female/ male	AdultAge/y	BMI/ kg m^−2^	Interview 1 (n = 16) 2 (n = 10)	Demography: Marital status, resident children	Socio-economy; living condition	Education level, employment,
1	F	59	43	1 + 2	Single	Urban townhouse	Medium-level education, early retirement
2	F	27	40	1	Cohabiting partner	Rural house	Medium-level education, on sick leave,
3	F	35	42	1 + 2	Single	Urban apartment	High-level education, full-time employment
4	F	55	46	1 + 2	Single	Urban apartment	Medium-level education, unemployed
5	F	41	48	1	Married, ≤ 2 children	Urban house	Medium-level education, on sick leave.
6	F	30	40	1 + 2	Single, ≤ 2 children	Urban townhouse	Low-level education, unemployed/on sick leave
7	F	30	40	1 + 2	Married, ≥ 3 children,	Urban apartment	Low-level education, unemployed
8	F	53	40,5	1	Single	Urban apartment	Middle education, full-time employment
9	M	53	41	1 + 2	Single	Urban house	Low-level education, early retirement
10	M	49	42,4	1	Cohabiting partner	Urban apartment	Medium-level education, part time employment
11	M	60	44,5	1 + 2	Married	Rural farm house	Medium-level education, full-time employment
12	M	42	55	1	Single	Urban house	Medium-level education, full-time employment
13	M	68	46	1 + 2	Single	Urban apartment	Medium-level education, retired
14	M	39	43	1	Single, ≤ 2 children	Urban townhouse	Low-level education, unemployed
15	M	45	44	1 + 2	Married, ≥ 3 children	Urban rented house	Low-level education, unemployed
16	M	25	47	1 + 2	Single	Urban 1-room rented apartment	Low-level education, unemployed

Low-level education refers to lower-secondary education; medium-level education refers to post-secondary education; high-level education refers to university college/tertiary education.

For the second interview five females and five males participated due to drop-out for the following reasons: giving birth, grief, no interest, no time, bariatric surgery and one was unreachable.

The interviews were carried out in 2016 by the first author and each interview lasted about 60 min. All participants spoke native Danish. Thirteen of 16 participants chose to be interviewed in their own homes and three participants chose a local hotel/cafe, a library or a nursing home. All interviews were audio-recorded and field notes were written immediately after each interview.

The interviewer was a non-obese, female PhD student. She was trained in conducting therapeutic dialogues and experienced in working with patients living with severe obesity due to years as a physiotherapist in the Department of Lifestyle Rehabilitation.

### Ethical considerations

The ethical principles were met through oral and written information about the project to the participants. Potential participants were called by the first author to clarify their interest in participating in the study. Information regarding voluntariness and the right to refuse participation in the study or to withdraw at any time during the project without it affecting their treatment was provided. Written and signed consent forms were conducted. Removal of identifying information and allocation of pseudonyms to each participant in the transcribed material and reports were ensured (World Medical Association, 2002). In the debriefing, the participant’s were provided with contact information in case of negative emotional responses after the interviews.

### Data collection and analysis

Individual face-to-face interviews were conducted in two rounds: prior to lifestyle intervention and again after 6 months. By repeating the interview, issues from previous interviews were clarified and deepened in the second interview as participants were asked to respond to key statements in the past interview (Kvale, 1996).

Pre-established interview guides consisting of open-ended questions related to a theory of lifeworld domains (Galvin & Todres, 1953–2013) were developed by the first and last authors for facilitating the interview. The first interview guide had its original focus on facilitators and barriers to PA based on a literature review (Toft & Uhrenfeldt, 2015) and perspectives of well-being and suffering in relation to being active (Galvin & Todres, 1953–2013). The second interview guide included clarifying questions from the first interview round and preliminary interpretation by the first author to be elaborated in greater depth by participants, so as to gather rich descriptions and illustrations. Briefing and debriefing were conducted during each interview to assess if interviews had done any harm to the participants (Brinkmann & Kvale, 2014). All interviews were transcribed verbatim.

The data analysis was performed step-wise based on the methods of Brinkmann and Kvale (2014) (See Table III) for each interview round (Brinkmann & Kvale, 2014). The analysis of the first interview round informed the second interview round as a preliminary interpretation of all interviews were used as background for further exploration in the second interview round six months later (Brinkmann & Kvale, 2014).

1. The 16 interviews of the first interview round were listened to and transcripts were read through several times to get a sense of the whole. Summary narratives for each of the participants were made by the first author to capture the breadth and sense of the whole in order to pursue a sense-making overview and to notice overarching patterns.
2. A process of identifying natural meaning units as expressed by the participants was undertaken for each transcript. After identifying the meaning units, they were condensed into more and more essential meanings. It entailed an abridgement of the words, but included the formulation of more general expressions of the essence of the meaning. Long statements were compressed into briefer statements, while the depth of description was enhanced.
3. A reflection of the transformed meaning units in terms of the aim of the study and the research question was undertaken to ensure that the meaning units were related to experiences of being active. Steps 1, 2 and 3 were conducted by the first author and discussed with co-authors.
4. Interpretation was a process that drew on the authors’ knowledge of lifeworld domains in order to grasp descriptions of well-being or suffering experiences, some of which were implicit, while others were explicit. The first author engaged in additional steps of reflecting on philosophical informed theory and the findings (Lindberg et al., 2016). In steps 5 and 6, the interview findings were merged into a whole, from which sub-themes and themes were developed.

**Table III.** Example of the step-wise analysis process

Interview round 1	Interview round 2				
1) Listening and reading interviews and making summery narratives.	2) Interview guide with additional clarifying questions based on the first interview round and preliminary interpretation by first author. Listening and reading interviews.	3) A reflection of the meaning units in relation to the aim of the study.	4) Interpretation based on lifeworld experiences of well-being among participants.	5) The reflective development of sub-themes and themes (Meaning for well-being or suffering).	6) The essential meaning of the participants’ voices (Dimensions of experience inspired by framework).

The natural meaning units were transformed in the analysis process to become descriptions of what it means for well-being and suffering and the new dimensions of experiences (See Table IV). Steps 4–6 were conducted in collaboration with the second and last author.

**Table IV.** Examples of meaning units, transformed meaning units, meaning for well-being, suffering and dimensions of experiences

Meaning unit	Transformed meaning unit	Meaning for well-being and suffering	Dimensions of experiences
“I don’t want to show myself … I just didn’t feel like taking of all my clothes when having fun under the sheets” Female #6, before intervention”	Feeling unattractive brings feelings of embarrassment and avoidance of sexual activity	Sense of being thwarted and defeated	Living within a downward spiral
“When I’m not in so much pain so I can start working out, because that’s what it’s going to take for me to lose weight and to make it less difficult for me to move around with my disabilities. I feel like it catches up to you. And that circle is so hard to break, because it’s so difficult doing it on your own” Female #1, before intervention	Pain limits the possibility of being active and losing weight		
“I can get a little angry with myself … get that feeling … you’re so lazy or you’re so stupid. That puts you in a bad mood which also takes the fun out of working out” Female #3, before intervention	Blame, mood and exercise are interacting negatively	Tackling energy depletion and sense of self	
“I’m very nice, to others, and think more about others than myself. I should think a little more about myself … I put others first and then myself last when I run out of energy. And then I don’t do anything for myself” Female #6, before intervention	Caring for others takes up energy for caring for oneself		
“It means a lot that I get beaten that little voice in my head that says: Well, you can**’**t do that, you**’**re too big**”** Female #3, before intervention	Fighting one´s inner voice of blame is comforting	Hoping for renewal and energized resoluteness	Striving for enjoyment and settlement
“It gave me a feeling of happiness to go for a walk and I could do that **…** wow, it feels good to get moving. It gives a sort of rush through the body almost like a high. It**’**s nice to realize how rewarding a simple walk is. That nice feeling of joy having moved your body and it gives you such a positive state of mind**”** Male #16, during intervention	Feeling physical capable brings a feeling of improved mood and vitality		
“I have to or I will never get on. It is kind of difficult sometimes to get everything sorted, but I am trying**”** Male #15, during intervention	Feeling obligated to keep trying despite the struggle	Enduring discomfort and feeling safe	
“Maybe it**’**s the fear of everybody looking at you. Maybe that**’**s what**’**s in the back of your mind and it**’**s bigger than you**’**d think. I have to break down some barriers if I want to reach my goal**”** Male #16, before intervention	Discomfort of others**’** judgement must be accepted		

### Findings

The findings are organized into two main dimensions of the participants’ experiences of being active, which are summarized in Table IV as “Living within a downward spiral” and “Striving for enjoyment and settlement”. The themes describing the experiences related to suffering were: ‘Sense of being thwarted and defeated “ and ‘Tackling energy depletion and impact of sense of self’. In relation to experiences of well-being the themes were: ‘Hoping for renewal and energised resoluteness’ and ‘Enduring discomfort and feeling safe’”.

Before intervention, the participants felt they were “Living within a downward spiral” and during the 6 months of intervention the participants were “Striving for enjoyment and settlement” and they experienced a general improvement of existential well-being in relation to being active.

### Living within a downward spiral

Before intervention, all participants pointed to PA as an effortful fight including existential challenges. The interrelatedness of their experiences was compared to a downward spiral including suffering characterized by feelings of homelessness, powerlessness, hopelessness and loneliness.

#### “Sense of being thwarted and defeated”

The experiences of suffering were related to the participants*’* sense of being thwarted and defeated in doing PA. They felt a kind of homelessness in doing PA, which was related to feeling alienated in different settings, incapable of mobilizing energy and bodily limited. They would avoid participation in activities, which were considered uncomfortable, unendurable or impossible, even though PA was thought to be a source of well-being in the long run. This self-reinforcing mechanism of an inactivity spiral was pulling them away from where they wanted to go.

As I’ve gotten bigger, those movements almost always cause discomfort. So it’s like everything’s connected, so that you don’t go and then you just get even fatter and then it’s even more difficult getting out the door because it’s hard … as you get bigger it becomes more of a hassle. Female #8, before intervention.

The feeling of being unable to live an active life influenced mood and energy negatively and added to the feeling of living in a vicious circle defined by lacking achievements without the counterbalance of well-being experiences. Little effort was put into their own well-being and others’ well-being was prioritized, which made their activity level go down, all the while a feeling of being a failure arose. Avoiding feelings of failure was considered important, as failures were found hard to handle, and an accumulation of failures made it even harder.

Then I just kind of give up. Well, you get to a point when you think what’s the point, it dosen’t matter, I might as well be fat for the rest of my life. It annoys me, it makes me angry, sad and then you just don’t have the energy to get out the door. It’s important for me not to have too many defeats. It means a lot to me anyway, not to have too many defeats, because it’s very hard for me to handle and to stick to [PA] There are long periods of time where you don’t do anything because you feel like you’ve failed and the reason for that failure is in fact myself. Female #3, before intervention.

In everyday life before intervention, a lot of attention was paid to weight, body size and bodily deficiencies, which were experienced as limitations regarding different everyday tasks, i.e., dressing, bathing, wiping oneself, cooking, cleaning, shopping, getting up and down from the floor, picking things up, walking, cycling and walking stairs.

It is to be complete honest hell, you know. It’s like my body doesn’t respond to me anymore. Female #1, before intervention.

The excerpts of data illustrated how feeling thwarted and defeated influenced activity level due to blame and energy depletion, and in some it would influence their eating pattern, weight development and sense of self.

#### “Tackling energy depletion and sense of self”

Part of being in a process of lifestyle change was the challenge of tackling energy depletion and the impact of sense of self.

Feelings of fatigue due to worries of one’s life situation, e.g., finances, health and relations to others were described as suffering, which took up energy and blocked the participants’ possibilities for moving forward and taking care of themselves. Being unable to re-energize by doing meaningful activities invoked boredom and isolation, which led to feeling irresponsible as well as feeling irritation, guilt and lack of respite. Moreover, feeling weighed down and depressed blocked action and added to the feeling of powerlessness and hopelessness in relation to making changes to one’s life, which had made some consider giving up on PA. Additionally, different kinds of fear, e.g., suffering defeats, losing control, becoming dependent, being ridiculed or embarrassed, (re)gaining weight, getting ill or dying were adding to the movement from lack of well-being into an existential suffering.

The lack of well-being was related to feeling incapable of putting sufficient efforts and performance in PA as well as losing weight. The experiences of defeats and lacking energy decreased their complacency and added feelings of blame and shame, which also brought sadness and frustrations. Covering up for what was considered one’s deficiencies was described as spending energy on being dishonest towards oneself and others. This was enacted by pretending to be something you are not in order to cover the feeling of being a human failure. The negative sense of self added to energy depletion and a sense of hopelessness in one’s own situation.

An existential suffering from hopelessness had before engaging in lifestyle intervention made three participants consider committing suicide.

Well, I did wonder whether it made a difference or if I should just give up … I did strongly consider that option … It’s just a matter of eating yourself to death, to be frank. And that’s all I’m going to say about that, to be honest. Male #9, before intervention.

Embodied discomfort was experienced when having difficulties in finding rest and relaxation and in the sensation of heavy breathing, sweating and vibrating fat when active. Bodily limitations were experienced as restriction in mobility and freedom of attending cherished activities such as playing, hunting, fishing or walking with others. The combination of reduced mobility and pain were bringing a feeling of incapability, which required cancellations of activities and brought the feeling of letting others and oneself down. Moreover, the large body was adding a feeling of being judged as different and wrong when being active.

…I was bloody dying and gasping for air, I just couldn’t keep up and that was embarrasing. It doesn’t take much when you’re that big … before you feel defeated … it’s hurtful in some ways. Male #14, before intervention.

Bodily dissatisfaction and negative body image would make some feel like an unwelcome stranger and keep them from entering a health club or a swimming pool or seek new social relations. Others would try to cover their body in clothing, e.g., when going out or having sex. Comparing their looks and performance to others was confirming the feeling of not fitting in. This constant awareness of self every waking hour of the day was considered a burden of embarrassment and brought a sense of inferiority that added feelings of sadness and shame. Avoiding or being unable to participate in physical and social activities made some isolate themselves at home, which added a feeling of loneliness.

Yet, being aware of their downward spiral, the participants felt powerlessness in regard to breaking the spiral pattern by themselves.

It’s a down-going spiral, I think or a vicious circle more specifically. Everything is connected in a greater game, I think. It’s easier to chose the unhealthy option about what to eat when you have those disabilities. Male #16, before intervention.

The suffering of homelessness, powerlessness, hopelessness and/or loneliness had initially led them to seek lifestyle intervention in order to break the negative patterns.

### Striving for enjoyment and settlement

By striving for enjoyment and settlement some participants found new possibilities of well-being, which counterbalanced their experiences of suffering. The spiral pattern described by the participants seemed to become merely a variation of interrelated and interacting experiences of well-being and suffering. The participants experienced increased freedom and cheerfulness in life as well as less focus on their limitations. This was related to improvements in; bodily abilities, social relations and insights into their own possibilities for well-being. All the while, there would still be suffering from depression, pain, and loneliness. The experiences of well-being were related to their hope for renewal and an energized resoluteness, as well as acknowledging the need for enduring some discomfort in order to become active.

#### “Hoping for renewal and energised resoluteness”

A meaningful life project was mainly to lose weight, and thereby, either becoming the parent, spouse, grandparent, friend or colleague they wanted to be. All participants wanted to be in relationships, they wanted to be somebody to someone. Becoming active was considered the means of becoming healthier and independent in order to preserve or regain a positive sense of self. However, struggling with guilt was commonly experienced in blaming oneself for acting irresponsibly by gaining weight and not exercising enough. During lifestyle intervention, participants began to put less emphasis on weight as the overall most important achievement.

I haven’t lost a huge amount of weight BUT I feel much better both mentally and physically and I have more energy and motivation. It’s gotten easier to manage things and I bike to work every day. I was really good at beating myself up and bringing myself down and I’ve stopped doing that. I have a strange sense of calm, so I’m taking it one day at a time and baby steps. I’ve stopped pressuring myself to achieve things that I want to achieve. I’ve found other ways to keep my spirits and my energy up. Female #3, during intervention.

Experiences of improved mobility, health, work life, hobbies, social activities and appearance as well as improvements in mood, energy and sense of self became valued and desired changes. Doing meaningful things in everyday life brought a feeling of contentment with the state of being as well as feeling empowered and energized to make changes. Others did not find the renewal they had sought in weight loss, which consolidated their feeling of failure, although healthier eating, improved sleep patterns and increased energy and activity level in everyday life were achieved.

I have done all that I possible could do, really everything, but I have not achieved anything—nothing has happened. Well, I would like to keep trying to do what I can by eating and exercising and all that, but I sometimes I feel that it is like digging a hole, to keep digging and digging without any end. Female #7, during intervention.

Due to lack of weight loss, she doubted if she would be able to stay in a continuously fruitful process on a long-term basis. Like others, she had not found enjoyment or settlement with being physically active. However, others had found positive expectations and hope for the future in relation to their activity level, relationships betweeen others and oneself.

#### “Enduring discomfort and feeling safe”

Being active was always to be considered in relation to the willingness to endure the discomfort of others glances when getting undressed to go swimming, having sex or a massage or feeling the bodily responses to movement.

I don’t like to show that much of my body, because I know that I have weight issues. And looking at my body makes me insecure … you don’t want to expose yourself. I just have this feeling that people are going to stare at me, more than they stare at other people Female #2, before intervention.

During intervention, some participants challenged how they could endure discomfort by seeking new experiences. Some were exercising vigorously and found it brought bodily well-being afterwards and a good conscience. Others would suffer pain and insights of their own bodily deficits. However, being able to endure some bodily discomfort brought optimism, positive expectations and cheerfulness for one’s future possibilities of being active. In relation to experiences of well-being, several enjoyable activities were mentioned that would bring the feeling of freedom and being alive, e.g., outdoor walks and bike rides. Bodily movement was desired and valued by some, especially at an individually adjusted pace. Letting go of pressure was a relief and the identification of possibilities for doing enjoyable things brought energy, vitality and the desire to do more of the pleasant things, e.g., some prefered going for walks alone in nature to think and reflect, others appreciated being with peers to attain the comfort and understanding of people with similar body conformation and mobility. Being alone or with peers eliminated the feelings of the shame and blame and was found encouraging.

It’s confidence or selfesteem that has been established by meeting people that walk around feeling the same as I do. It has just given me some kind of confidence boost that gives me the courage to stand up for myself, because sometimes you can feel a little ashamed that you aren’t able to just pull yourself together, but you can’t just do that. It’s not just about pulling yourself together. There are so many factors at play and they understand that Female #3, during intervention.

Moreover, the females emphasized how being with pets provided a feeling of safety and respite as it made it unlikely to be bullied or discriminated.

The process of undergoing lifestyle intervention was found a be a process of acting on the possibilities appearing and to settle with the limitations.

### Discussion of findings

The aim of this study was to explore and describe adults’ existential experiences of being active when living within a large body before and during lifestyle intervention. It contributes to new descriptions of the dynamics and interchangeability of well-being and suffering. The existential experiences of powerlessness, hopelessness, homelessness and loneliness were interrelated to PA participation and counterbalanced by well-being experiences. Experiences were temporal and changed over time with new experiences. This concurs with previous findings of how ambivalent feelings towards PA were based on shifting experiences (Danielsen et al., 2016).

Before intervention, the participants of our study were mainly suffering from powerlessness and hopelessness of being caught in an inactivity spiral. Similar aspects of hopelessness in relation to weight loss have also been described among less obese individuals undergoing group treatment in primary care practice (Östberg et al., 2011) and the downward spirals have previously been described in relation to how severely obese individuals explained the development of their obesity (Owen-Smith et al., 2014). However, this study describes how the massive existential suffering in more of the lifeworld domains was experienced and led three participants to consider giving up and committing suicide. This may be equivalent to losing the horizon of future possibilities and only seeing hopelessness in the present situation (Galvin and Todres, 1953–2013).

Suffering from a kind of homelessness was reminiscent of loss of previous capacities and identities, and bodily responses of pain and discomfort when being active. We found that avoiding physical and social activities may be due to feelings of shame, blame and lowered mood, and that withdrawal from the world was a way to protect one’s vulnerabilities. According to previous reflective lifeworld research on loneliness, such withdrawal may add to one’s existential suffering (Dahlberg, 2007).

Fighting, settling and giving up were central aspects. Exercise and weight loss were a fight and the exhausting alternative was to give up. Finding settlement in one’s present situation seemed difficult for those disliking themselves and their lives, which prevented them from feeling “at home” while being active (Heidegger, 2001). But giving up on being active would be equivalent to giving up upon oneself and one’s future, which no one did. Therefore, being active was of great importance and required great efforts.

Being able to find one’s own way round PA is based on individual values and possibilites, rather than living as one ought to do by drifting along with the others’ choices of living (Sarvimäki, 2006). Being able to achieve this may be based upon insights developed through dialogues and experiences with others by mirroring oneself in the otherness as something complementary to one’s own lifestyle (Gadamer et al., 1992).

The findings point to possible understandings of well-being, which may relieve suffering and increase the patients' feelings of being capable (Lundqvist et al., 2002). In particular, it seems important to strive for settlement in one’s current situation as well as seeking future possibilities for vitality in order to experience well-being, and/or to become more active (Dahlberg et al., 2009).

The person-oriented perspective adds depth to previous research, which has identified barriers to PA among severely obese individuals to be; body dissatisfaction, lack of self-efficacy and reduced mental health, whereas facilitators were; enjoyment, positive body image and supportive active relationships (Dikareva et al., 2016).

#### Discussion of design and methods

The longitudinal design was found appropriate to explore the temporal dimensions of the participants’ experiences of being active over the 6-month period the intervention programme lasted, i.e., in relation to identifying changes and continuities (Balmer & Richards, 2017). This study is the first step, which was planned to be followed by a third interview round 12 months later. A strength of the repeated interviews was the prolonged engagement with the participants, which provided time to establish a trustful relationship as well as for hermeneutic reflection between the interviews where continuous discussions with the research team were conducted (Gadamer, 2013). This was found to increase the trustworthiness of the findings (Fleming et al., 2003).

The sampling size was considered to provide sufficient data to gain a better understanding of the subject matter (Sandelowski, 1995) and the equal representation of two genders was also considered a strength. However, a weakness of this study may be that not all participants underwent the intervention as planned: one female was physically incapable of attending and another female cancelled the intervention after the first admission. However, this means that the findings of the study represent not only the perspectives of severely obese individuals with the capacity to engage with the healthcare system, but also the perspectives of participants, who were not capable of undergoing the lifestyle intervention programme. But, it may also mean that it was the most vulnerable patients who dropped out. Moreover, it may be considered a weakness that only ethnic Danes were included and only their perspectives are presented. However, this was due to the fact that no immigrants were available at the time of inclusion for the study.

#### Discussion of theory and methodology

Existential philosophy was found appropriate in relation to the aim of this study as it is individual and unique and developed through the participants’ “world of experiences” (Hörberg et al., 2019). The lifeworld approach provided insights into each participant's insider perspectives of what it was like for them to be physically active (Todres et al., 2014) and the lifeworld domains were useful for the philosophical examination and the general discussion of findings after the empirical analysis (Lindberg et al., 2016).

In the literature, well-being has been defined differently, e.g., health, quality of life or the subjective experience of feeling good and being satisfied (Sarvimäki, 2006). These definitions tend to divide well-being into separate areas, i.e., psychological, physical, social or spiritual. We approached human well-being in the ontological understanding of being well in a seamless everydayness (Sarvimäki, 2006). We found the conceptual framework suitable for researching well-being as a unity (Galvin & Todres, 1953–2013). Moreover, the conceptual framework provided a holistic and person-centred approach (Galvin, 2010) and helped to identify ways of enacting a humanized care in practice (Todres et al., 2009) with the purpose of making the patient feel like they are “being met” (Dahlberg et al., 2009).

Another strength is that the lifeworld theory is specifically used in a Danish context, in a lifestyle intervention setting and with participants living with severe obesity. Previously, the conceptual framework (Galvin & Todres, 2011) has mainly been used in the UK (Mayoh & Jones, 2015; Shaw et al., 2016) or in different settings or populations in Denmark (Uhrenfeldt & Hoybye, 2015; Rasmussen et al., 2018). However, a recent Norwegian study has explored how people with obesity struggle to gain well-being in life (Haga et al., 2019a).

A weakness may be that qualitative research interviewing is not neutral, as it may have affected the participants' thoughts and actions (Dempsey et al., 2016), even though it was not the purpose.

The findings reported are partial (fuller findings are reported) (Toft, 2019). The present paper does not claim to be an analysis of the whole phenomenon, but rather aims to illuminate the depth of insight regarding the lifeworld experience of people who live in a large body. The new insights revealed are led by what participants described and are sensitized by new theoretical ideas about well-being.

## Conclusion

Patients living with severe obesity may face numerous interacting existential experiences, which can be considered facilitators or barriers to PA. Based on our findings, we consider it relevant for HCPs to address the individual’s lifeworld experiences of well-being, lack of well-being and suffering in their being and doing. Well-being as a sense of feeling “at home” when physically active may break down an inactivity spiral. Promoting well-being is a legitimate aim of lifestyle intervention as existential suffering reduces the possibilities of changing lifestyle.

### Relevance to clinical practice, education and research

In clinical practice, it seems important to rethink treatment of patients living with severe obesity to ensure that they will benefit and not be harmed by suffering defeats when attending lifestyle intervention. Standard recommendations for exercise and weight loss may add to a sense of defeat and actual defeat if not adjusted to the individual’s lifeworld. New insight into the well-being and suffering spirals may be helpful in supporting patients in discovering their own possibilities to increase well-being and to avoid overemphasizing weight loss in ways that are counter-productive. Interventions may emphasize the benefit of finding settlement to improve the ability to engage in meaningful living. In this way, the existential perspectives may offer an ethically sensitive and respectful approach to PA promotion.

Furthermore, a philosophically oriented theoretical foundation, drawn from a lifeworld perspective can serve as a coherent direction for caring practices in education and may tie a traditional fragmented approach together in a greater unity, offering a more holistic framework for care.

This study points at further research into future investigations on the interaction between patients and HCPs in order to balance well-being and suffering experiences.
